# *Bacteroides*, butyric acid and *t10,c12-CLA* changes in colorectal adenomatous polyp patients

**DOI:** 10.1186/s13099-020-00395-0

**Published:** 2021-01-12

**Authors:** Ciyan Chen, Min Niu, Junxi Pan, Na Du, Shumin Liu, Huanqin Li, Qiuyue He, Jian Mao, Yong Duan, Yan Du

**Affiliations:** 1 Department of Clinical Laboratory, the First Affiliated Hospital of Kunming Medical University, 295 Xichang Road, Wuhua District, Kunming, 650032 China; 2 Yunnan Institute of Laboratory Diagnosis, Kunming, 650032 China; 3 Yunnan Key Laboratory of Laboratory Medicine, Kunming, 650032 China; 4 Qujing Healthcare Security Administration, Qujing, 655000 China; 5 Department of Clinical Laboratory, the No. 1 Affiliated Hospital of Yunnan University of Chinese Medicine, Kunming, 650032 China

**Keywords:** Colorectal adenomatous polyps, Microbiome, 16S rRNA, SCFA, Bile acid, CLA

## Abstract

**Background:**

Colorectal adenomatous polyps (CAPs) are considered precancerous lesions of colorectal cancer (CRC). The gut microbiota participates in the process of digestion and, in the process, produces metabolites, mainly short-chain fatty acids (SCFAs), secondary bile acids and conjugated linoleic acid (CLA). This study aimed to investigate the gut microbiota constituents and metabolites in the faeces of CAP patients to identify microbiota or metabolites that can be used as sensitive biological predictors and to provide a theoretical basis for the clinical treatment of CAPs.

**Methods:**

16S rRNA sequence analysis was used to detect microbial changes in the faeces of CAP patients. qPCR analysis was used to evaluate the ability of the microbiota to produce metabolites, and the contents of metabolites in faeces were detected by ion chromatography and ultra-performance liquid chromatography-tandem mass spectrometry (UPLC-MS/MS).

**Results:**

Based on the detection of the gut microbiota, patients with CAPs had increased abundances of *Bacteroides* and *Citrobacter*, and the abundances of *Weissella* and *Lactobacillus* were decreased. We also explored gene expression, and the abundance of butyrate-producing bacterial genes was significantly increased in the faeces of CAP patients, but those of secondary bile acid-producing and CLA-producing bacterial genes showed no differences in faecal samples. The acetic acid and butyric acid contents were increased in the faeces of the CAP group, and the healthy control group had higher *t10,c12-CLA* contents*.*

**Conclusion:**

The gut microbiota analysis results, assessed in faeces, showed that *Bacteroides* and *Citrobacter* were positively correlated with CAPs, which indicated that changes in specific genera might be detrimental to intestinal health. In addition, *t10,c12-CLA* played an important role in protecting the intestine.

## Background

Colorectal cancer (CRC) is a common malignant tumour of the digestive tract, with the third highest incidence and second highest mortality among tumours worldwide [1, 2]. Colorectal adenomatous polyps (CAPs) are regarded as a critical precursor to CRC [3], and adenoma is an early neoplastic tissue that has not gained the properties of a cancer.

There are 100 trillion bacteria in the human intestine, and the collective genome of these bacteria is called the gut microbiome, which is 150 times the size of the human genome [4]. The gut microbiota is essential for the growth and physical health of the human body. The gut microbiota participates in digestion and absorption of food in the intestines and actively participates in cell-mediated immune responses, which maintain intestinal barrier function and intestinal environment stability. The imbalance of this symbiotic relationship might have an adverse effect on the host, and gut microbiota imbalance has been observed in cases of inflammatory bowel disease (IBD) [5], obesity [6], ageing [7] and cancer [8].

Moore et al. [9] applied culture methods to analyse the faeces of CRC patients and colorectal polyp patients and found that the abundances of *Bacteroides* and *Bifidobacteria* were positively correlated with the risk of colon polyps, while those of *Lactobacilli* and *Eubacteria* were related to the intestinal tract and had a protective effect. However, the types of bacteria that could be cultured in faeces were limited, and most of the bacteria could not be cultured in an in vitro environment. Therefore, the emergence of high-throughput sequencing technology and metagenomic analysis provided a better solution for analysing complex microbiome data. *Fusobacterium* has been identified as a risk factor for both colorectal adenomas and cancer [10], and the mechanism of the *F. nucleatum* association with CRC has been clarified in mice [11].

Intestinal metabolites, such as short-chain fatty acids (SCFAs) and bile acids, are strongly linked with cancerous conditions in the gut [12]. Conjugated linoleic acid (CLA) is considered a health-promoting fatty acid, and the anti-cancer properties of CAL in vivo and in vitro have been widely recognized [13]. Some *Firmicutes* use the butyryl-CoA:acetate CoA-transferase route to produce butyrate, and the proportion of propionate presented in faeces correlated with the relative abundance of *Bacteroidetes* [12]. Owning to their generation of reactive oxygen species (ROS) and reactive nitrogen species (RNS), which cause DNA damage, bile acids have been implicated in carcinogenesis.

To investigate the gut microbiota profile in CAP patients, we collected faeces from CAP patients attending the First Affiliated Hospital of Kunming Medical University. High-throughput sequencing technology was used to analyse the gut microbiota in the intestinal tract, which extended the understanding of the microbial community. Analysis of the faecal metabolites of CAP patients was performed to identify the metabolites that changed and to explore the changes in the gut microbiota and its metabolites during the progression of CAP to CRC. This study will provide comprehensive information about the gut microbiota and metabolite changes in CAP patients, which will help to characterize the role of gut microbiota and metabolites in adenoma occurrence and progression, and the differences in the gut microbiota and metabolites might be considered biomarkers of CAP in the future.

## Methods

### Sampling

Thirty patients with colorectal adenomatous polyps were selected from the First Affiliated Hospital of Kunming Medical University from November 2017 to April 2018. Exclusion criteria included obesity, special eating habits, colorectal cancer, history of colorectal surgery, colitis (ulcerative, Crohn’s), metabolic disease (diabetes, hyperlipidaemia), and infectious disease. Thirty healthy volunteers were selected from the First Affiliated Hospital of Kunming Medical University as controls. No subjects were taking antibiotics, immunosuppressive agents, corticosteroids or probiotics within 3 months prior to sample collection. There were no significant differences in age, gender, or BMI between the two groups (*P* > *0.05*) (Table 1). Stool samples from volunteers were preserved after admission, and samples were collected in accordance with the relevant guidelines and regulations.

**Table 1:** Demographic information

	CAP (n = 30)	HC (n = 30)	P-value
Age	53.23 ± 10.14	50.33 ± 10.87	0.287
Gender (male/female)	20/10	13/17	0.069
BMI (kg/m^2^)	24.77 ± 2.00	24.48 ± 1.83	0.451

### DNA extraction and 16S rRNA gene amplification

Genomic DNA was extracted from stool samples and biopsy samples with the QIAamp DNA Stool Mini Kit (Qiagen, Hilden, Germany). The primers for amplification of the V3-V4 region of the bacterial 16S rRNA gene, after amplification high throughput sequencing was performed with the Illumina MiSeq platform (Illumina, CA, USA).

### Bioinformatics analysis

The high-quality paired-end reads were combined to tags based on overlaps, and the consensus sequence was generated by Fast Length Adjustment of Short reads (FLASH, v1.2.11). The tags were clustered into operational taxonomic units (OTUs) by scripts in USEARCH (v7.0.1090) software, OTUs were clustered with a 97% similarity cut-off using UPARSE, and OTU representative sequences were taxonomically classified using the Ribosomal Database Project (RDP) Classifier v.2.2 trained on the Greengene_2013_5_99 database with a 0.6 confidence value as the cut-off. OTUs were filtered by removing unassigned OTUs and removing OTUs not assigned to target species.

OTUs were used for α diversity estimation. Comparison of the β diversity, which is the difference in species diversity between two groups, was performed based on the OTU abundance by QIIME (V1.80).

Specimen annotation analysis is a method that compares OTUs to a database of classified OTUs at the phylum, class, order, family and species levels, and the analysis is then presented by histograms. UniFrac analysis used phylogenetic information to compare species community differences between samples.

### Metabolite determination

#### Ion chromatograph analysis

The faecal samples stored in the refrigerator at − 80 °C were removed, weighed to 300 mg, dissolved in 1 mL of dH_2_O, vortexed and mixed for 30 s. Then, the supernatant was incubated with a 0.22 μm microporous nylon membrane (water system). The liquid was filtered, placed in an EP tube and placed in a refrigerator at − 20 °C for use. Standard curves were generated by using standard solutions. A volume of 25 μL was performed into chromatographic columns (DIONEX IonPac AG11-HC 4 × 50 mm & IonPac AS11-HC 4 × 250 mm, USA) and eluted with KOH at a flow rate of 1.2 mL/min; ions were detected by a conductivity detector in an ion chromatograph (Thermo Dionex ICS-3000, USA), and the column temperature was 30 °C.

#### UPLC-MS/MS analysis

The faecal samples stored in the refrigerator at − 80 °C were removed, weighed to 200 mg, dissolved in 1 mL of methanol, vortexed and mixed for 30 s. The supernatant was then incubated with a 0.22 μm microporous nylon membrane (organic system). The liquid was filtered, placed in an EP tube and placed in a refrigerator at − 20 °C for use. Standard curves were generated by using standard solutions.

#### UPLC conditions

The mobile phase consisted of 0.05% ammonia (5 mM aqueous solution) in water as solution A and acetonitrile as solution B. The flow rate of the mobile phase through the column was 0.4 mL/min (Waters BEH C18 1.7 μm, 50*2.1 mm, USA) at a temperature of 40 °C. The injection volume was 1 μL. Mass spectrometry conditions included electrospray ionization, negative ion mode, multiple reaction detection, air as the desolvation gas, nitrogen as the cone gas, and argon as the collision gas.

### Real-time PCR analysis

To explore bacteria that produce specific metabolites, real-time PCR was used (TIANLONG Gentier 96, Xi’an, China). The PCR primers used were as follows: 16S rRNA (forward, TGGAGAGTTTGACCTGGCTCAG; reverse, TACCGCGGCTGCTGGCAC); butyrate-producing bacteria, determined by the presence of the BCoA gene (forward, GAGGTCGCTTCTCTTTGTATGC; reverse, TCGTGTTGTGAAATGTTGGGTT); secondary bile acid-producing bacteria, determined by the presence of the BaiCD gene (forward, CAGCCCRCAGATGTTCTTTG; reverse, GCATGGAATTCWACTGCYTC); and conjugated linoleic acid-producing bacteria, determined by the presence of the PAI gene (forward, TTGGGGGCGTTATTTATGGTTA; reverse, TTACGTTAGTCAAACATCTTCTTAG). Real-time PCR experiments were performed with GoTaq Green Master Mix (Promega, WI, USA) in a total volume of 20 μL. The amplification cycle used was 1 cycle of 95 °C for 2 min; 45 cycles of 95 °C for 10 s, 60 °C for 30 s, and 72 °C for 1 min each with data acquisition at 72 °C; 1 cycle of 72 °C for 10 min; and cooling to 4 °C. To obtain the Ct value of samples, 2^−∆∆Ct^ was calculated for statistical analysis.

### Statistics analysis

T-test and Wilcoxon rank-sum test were used to analyse differences between two groups with SPSS 22.0, and results presented by GraphPad Prism 7.0. Difference with *P* < *0.05* were considered to be statistically significant.

## Results

### Changes in the gut microbiota in the faeces of CAP patients

A total of 9433 OTUs were generated, with an average of 168 OTUs per sample, and the library coverage of all samples was over 99.9%. Thus, the sequencing depth covered all the species in the samples.

The α diversity analysis indicated that the Chao, Ace, Shannon and Simpson indexes were not significantly different between the two groups (Additional file 1: Figure S1). To observe the difference in composition of the two sample types by principal coordinate analysis (PCoA) (Additional file 1: Figure S2), in which the PC1 coordinates represent the main coordinate component that caused the largest difference in samples, PC1 explained 21.4% of the difference, followed by PC2, explaining 12.01%. In the PCoA diagram, it was observed that the samples in the CAP group and healthy control (HC) group were not completely separated, and some samples were aggregated.

Analysis of bacteria at the phylum level**.** A total of 12 bacterial phyla were found in the CAP and HC samples. The phyla with the highest proportions in the two groups were *Firmicutes* (CAP, 52.07%; HC, 55.32%), *Bacteroidetes* (CAP, 26.93%; HC, 23.44%) and *Proteobacteria* (CAP, 20.52%; HC, 19.32%).

**Fig. 1:**
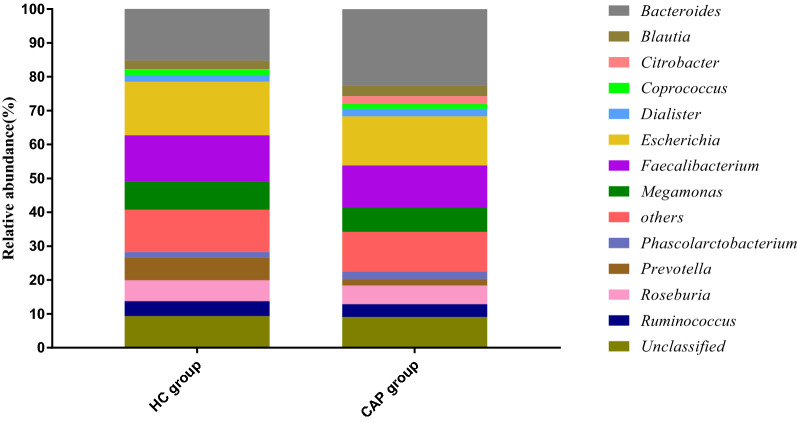
Composition of gut microbiota at genus level. Genus with relative abundance greater than 1% are presented. The lower genus are grouped as “Others”. *Prevotella* level was lower in the CAP group that in the HC group, but no significant differences were observed

At the genus level, a total of 121 bacterial genera were found in the CAP and HC groups, of which the highest proportions were *Bacteroides* (CAP, 22.51%; HC, 15.28%), *Escherichia* (CAP, 14.50%; HC, 15.83%) and *Faecalibacterium* (CAP, 12.45%; HC, 13.60%). Among all genera, the abundances of *Citrobacter* (*P* < *0.05*) and *Bacteroides* (*P* < *0.05*) increased, and the abundances of *Weissella* (*P* < *0.01*) and *Lactobacillus* (*P* < *0.05*) decreased in the CAP group (Fig. 1).

Weighted UniFrac analysis of species phylogenetic evolution. Differences in the diversity between the two groups of samples, including the weighted UniFrac analysis of OTU abundance and the observation of OTU abundance in a heatmap, were compared via β diversity analysis (Additional file 1: Figure S3). The distance between samples was changed, but the differences were not significant.

### Changes in metabolites in CAP patients

**Fig. 2:**
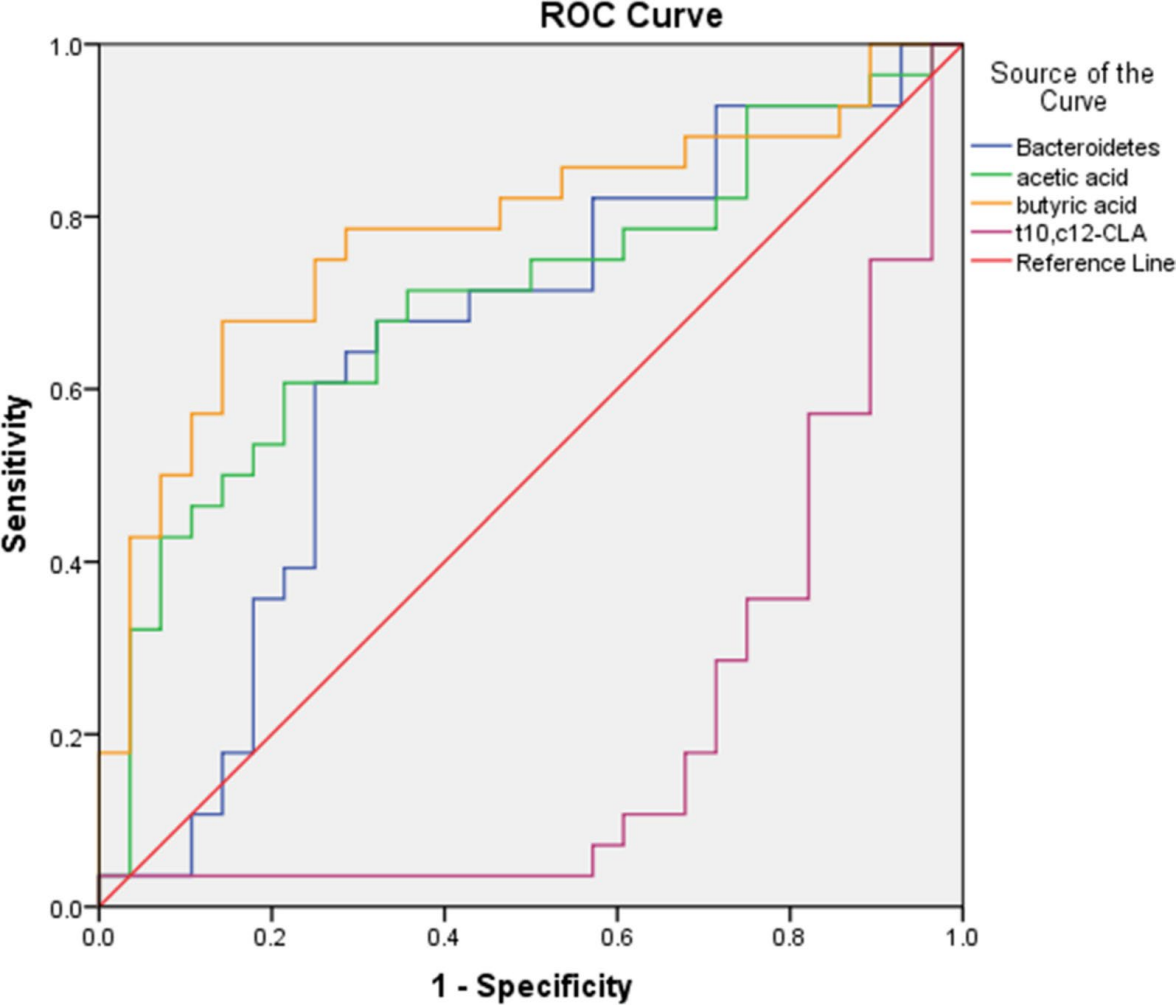
ROC curve describing the prediction accuracy of *Bacteroidetes*, acetic acid, butyric acid *t10,c12-CLA* these four particular genus and metabolites, with the AUC values 0.648, 0.704, 0.781 and 0.203 respectively. The AUC value above 0.9 with a higher accuracy, AUC between 0.7 ~ 0.9 with a certain accuracy, AUC value between 0.5 ~ 0.7 with a lower accuracy, AUC under 0.5 indicated with no diagnostic value

The SCFA, bile acid and CLA quantification results showed that acetic acid and butyric acid contents increased, while *t10,c12-CLA* content decreased in the faeces of CAP patients (*P* < *0.05*) (Table 2). A receiver operating characteristic (ROC) curve described the prediction accuracy of these three different metabolites and *Bacteroides*. The areas under the curve (AUCs) of *Bacteroides*, acetic acid, butyric acid and *t10,c12-CLA* were 0.648, 0.704, 0.781 and 0.203 respectively (Fig. 2). To explore these four particular genus and metabolic factors, the AUC values between 0.7 ~ 0.9 indicate certain accuracy, AUC values between 0.5 ~ 0.7 indicate a lower accuracy, and AUC values under 0.5 indicate no diagnostic value.

**Table 2:** Metabolites analysis and comparison of CAP patients and healthy volunteers

	CAP (mg/L)	HC (mg/L)	P-value
Acetic acid	596.24 ± 176.54	468.27 ± 171.63	0.003
Propionic acid	193.15 ± 81.10	166.60 ± 69.92	0.186
Isobutyric acid	10.49 ± 6.99	8.73 ± 10.85	0.090
Butyric acid	300.09 ± 186.32	143.87 ± 95.79	0.000
Isovaleric acid	8.72 ± 4.97	12.08 ± 14.92	0.941
Valeric acid	23.55 ± 20.47	25.11 ± 18.82	0.7902
CA	35.91 ± 67.93	39.38 ± 59.67	0.241
CDCA	36.51 ± 34.10	35.56 ± 49.02	0.22
DCA	52.61 ± 58.92	29.27 ± 32.22	0.22
LCA	12.52 ± 14.64	18.76 ± 24.15	0.45
*c9,t11-CLA*	108.96 ± 125.42	173.33 ± 200.50	0.234
*t10,c12-CLA*	11.28 ± 14.96	17.90 ± 13.06	0.013

### qPCR analysis of metabolites produced by bacteria

The dissolution curve and amplification curve of the internal reference gene and the target gene were observed to be complete, smooth and without peaks, indicating that the specificity of the amplification products was good.

**Fig. 3:**
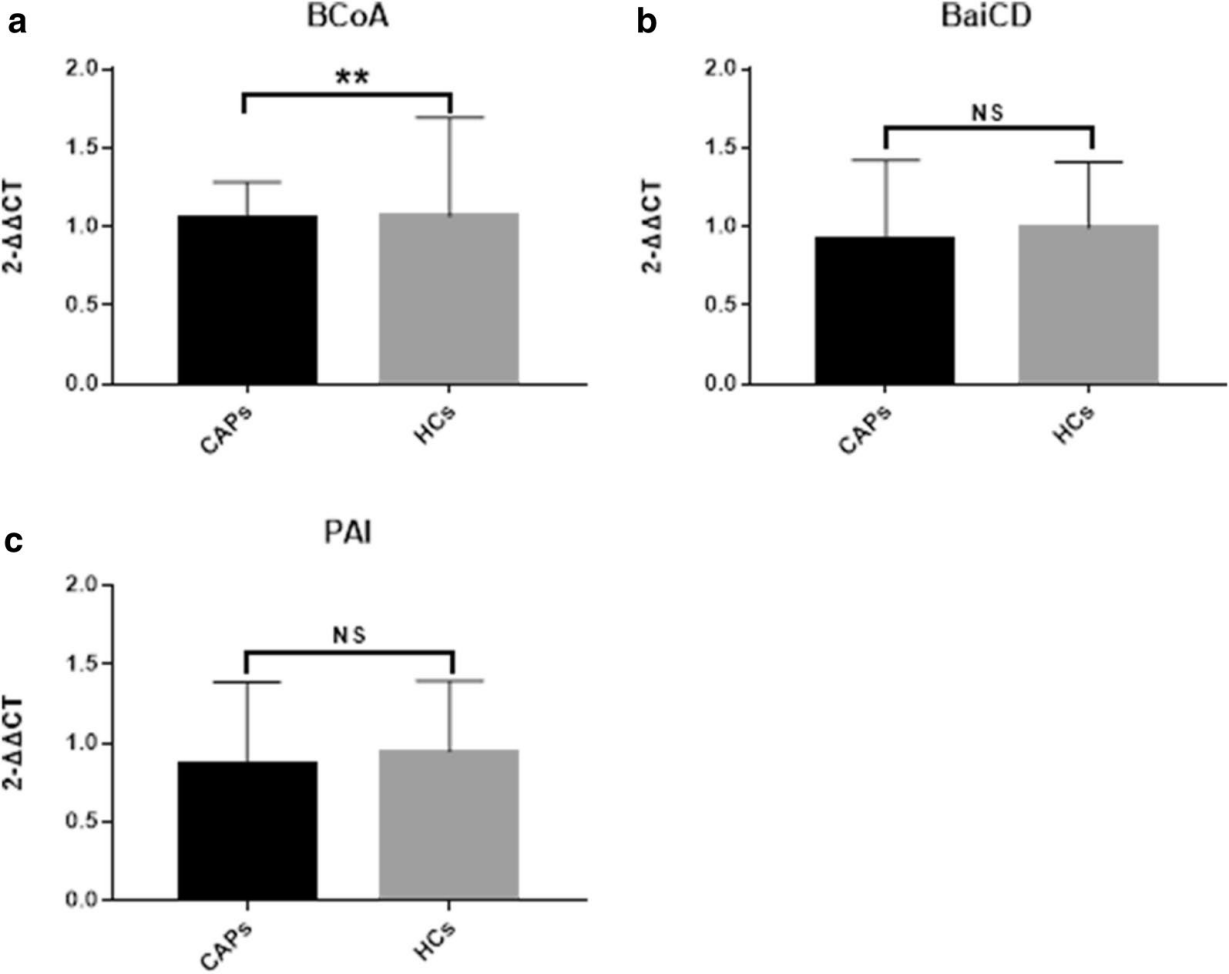
The detected gene expression of bacteria in faecal samples possessed the ability of producing metabolic, leading to comparison with 2-∆∆Ct. *NS* none significantly differences, **P < 0.05

In faecal samples, the gene expression of butyrate-producing bacteria in the CAP group was lower than that in the HC group, but the gene expression of bile acid-producing bacteria and conjugated linoleic acid-producing bacteria in the CAP group was not significantly different from that in the HC group (Fig. 3a–c).

## Discussion

Complex microbes inhabit the human intestine, and the group maintains the stability of the intestinal environment and protects the health of the human body. These microbes participate in defence and immunity against pathogens, development of intestinal microvilli, fermentation of nondigestible dietary fibres, and anaerobic metabolism of peptides and proteins, providing energy to the host. A number of studies have shown that the gut microbiome is associated with the occurrence of CAPs. However, most intestinal bacteria cannot be cultured in vitro, and the application of high-throughput sequencing technology helped us fully understand how the gut microbiome changed during the development process from healthy status to CAP and CRC.

In the 16S rRNA sequence analysis of faeces, the α diversity index was not significantly different between the two groups. Goedert et al. reported similar results for the faecal microbiota in CAP patients [14], but a reduction in the abundance of the faecal microbiota was observed in CRC patients [15]. PCoA of weighted UniFrac distance revealed no obvious aggregation in the CAP group and HC group. The changes in genera in both groups were analysed, and *Weissella* and *Lactobacillus* were present in the HC group; although their relative contents were not high, *Weissella* and *Lactobacillus* are probiotics [16]. The difference in the two groups indicated that the two genera might have a protective effect on the intestine. In addition, the abundances of *Bacteroides* and *Citrobacter* in the CAP group were higher than those in the HC group, which indicated that these two species might play an important role in the pathological process of CAP. Studies have found that *Citrobacter* can take over the cell–cell communication system to trigger colitis in mice [17], and the elevation of *Bacteroides* abundance in the faeces of CAP patients has been confirmed [5]. Compared with the bacteria in faeces, the bacteria attached to the colonic mucosa are more likely to affect the gene expression of colonic mucosa cells. Based on high-throughput sequencing of biopsy tissue [18], the α diversity of polyps was higher than that of healthy tissue, which indicated that polyps have higher within-habitat diversity than healthy tissue. This phenomenon of increased diversity also appeared in studies on CRC [19], which might suggest that increased diversity of the gut microbiome is not a sign of healthy intestines but rather the excessive growth of various harmful bacteria or archaea in adenoma and cancer development [3]. Studies on the faeces and tumour tissues of CRC patients have shown different results for *Bacteroides* [20–22]. Yu et al. [23] found that the abundances of *Proteobacteria* and *Fusobacteria* were high in tumour tissues of CRC patients, but in this study, there were no significant differences in the two groups of bacteria. Many studies have found that *Fusobacteria* were enriched in the faeces and tumour tissues of patients with CRC [24], but there were no significant differences in the abundance of *Fusobacteria* in faeces or adenoma tissues between CAP patients and HCs. The relative content of *Fusobacteria* in faeces and polyps was low, and it was speculated that the enrichment of *Proteobacteria* may be related to the degree of tissue abnormality.

Studies have indicated the relationship between the gut microbiota and metabolites in the intestinal tract. The ability of the gut microbiota to produce metabolites, such as butyrate, secondary bile acid and CLA, can vary with gut environment modulation, as has been shown in response to diet. This study aimed to investigate whether there were gene expression differences between CAP patients and healthy volunteers, and the acetic acid and butyric acid contents in the faeces were higher in the CAP group than in the HC group. Butyrate, as a major source of energy for intestinal epithelial cells, can reduce colonic inflammation, induce apoptosis, inhibit tumour cells and prevent CRC development. The anti-proliferative and anti-cancer properties of butyrate have been demonstrated and are probably attributable to the effect of high concentrations of butyrate as a histone deacetylase inhibitor (HDACi) [25]. However, Bultman et al. [26] believed that butyrate is a causative factor of CRC, and a study on APC^Min/+^MSH2^−/−^ mice fed butyrate showed that the amount of butyrate administered was positively correlated with polyp formation in mice, which might be due to the stimulation of gut microbiota hyperproliferation and mouse intestinal epithelial cell transformation through metabolites. Polyp formation at low concentrations stimulates colonic epithelial cell proliferation [27]. These opposing effects of butyrate have been called the “butyrate paradox”. Although the propionic acid content was not significantly different between the two groups, the propionic acid content in the CAP group was increased, and *Bacteroides*, which is a major contributor to propionate synthesis, was significantly more abundant in the CAP group. In the analysis of DNA from faeces, the expression of butyrate-producing bacterial genes in the CAP group was significantly lower than that in the HC group, but there was no significant difference in DNA between the two groups. The results indicated that the abundance of butyrate-producing bacteria in the faeces of CAP patients was decreased, while the butyric acid content in the CAP group was higher than that in the HC group. Ferrer-Picón Elena et al. observed that a lower stool content of butyrate-producing bacteria was not correlated with the butyrate concentration in IBD patients [28]. The faecal acetate and butyrate concentrations were positively correlated with supplementation with resistant starch and non-starch, which indicated that diet composition and intake influenced the actual SCFA concentrations in the gut [12]. Therefore, the faecal SCFA concentrations does not fully reflect the concentration of SCFAs produced by gut microbiota fermentation; thus, the intestinal health effects need to be carefully considered [29]. SCFAs can effectively reduce the intestine pH, promote glycolysis of food in the intestine and reduce carcinogen absorption, which can reduce CRC risk [30].

Due to the different positions and conformations of the conjugated double bonds, there are multiple isomers of conjugated linoleic acid, and the main isomers are *c9,t11-CLA* and *t10,c12-CLA* [31]. These two isomers play different roles in anti-cancer and anti-cardiovascular disease activity. Here, *t10,c12-CLA* content was found to be increased in the faeces of the HC group, but the difference in *c9,t11-CLA* content between groups was not statistically significant. CLA has functions such as reducing body fat, restricting tumour development, preventing cardiovascular disease and improving immunity [32]. As a fatty acid that protects the intestine, its anti-tumour properties in vitro and in vivo have been widely recognized [13]. Among the isomers, *t10,c12-CLA* has functions of reducing body fat, lowering triglyceride content and inhibiting adipocyte differentiation, and *t10,c12-CLA* was found to be more effective than other isomers in inhibiting tumours. The tumour cell growth inhibition effects were positively correlated with its concentration, and *c9,t11-CLA* played an important role in immune regulation [33–35]. In addition, *t10,c12-CLA* content showed a significant decrease in the CAP group, and the decrease in faecal *c9,t11-CLA* content might increase the risk of intestinal adenomatous polyps. Certain *Bifidobacteria* species in the gut, as natural colonizers, are capable of converting linoleic acid to *c9,t11-CLA*, *t10,c12-CLA* and small amounts of *t9,t11-CLA* [36]. There was no statistically significant difference in faecal *c9,t11-CLA* content between the two groups, and the abundance of *Bifidobacteria* that produce *c9,t11-CLA* was not significantly different between the two groups.

A high-fat diet strongly stimulates bile acid production, and bile acids are converted to secondary bile acids, deoxycholic acid (DCA) and lithocholic acid (LCA), after structural modification of bacteria with 7α-dehydroxylating activity in the gut. DCA is the most typical secondary bile acid [15]. Secondary bile acids promote the proliferation of intestinal epithelial cells, induce apoptosis and mutation, and promote cancer progression [37]. There were no significant differences in the levels of any bile acids in our study, but the DCA content was higher in the CAP group than in the HC group. Lu et al. [38] found that faecal chenodeoxycholic acid (CDCA) and DCA contents were significantly increased only in CRC patients, but no significant differences were observed in CAP patients. The gut microbiota converts primary bile acids into secondary bile acids, suggesting that the gut microbiota can affect the composition of secondary bile acids, while changing the secondary bile acid profile could reshape the intestinal bacterial composition [39]. Although there was no significant difference in DCA or CDCA content between groups, the abundance of *Bacteroides*, which has bile acid-resistant characteristics, was positively correlated with fat and protein intake [26, 40, 41], and the *Bacteroides* abundance showed a significant increase in the CAP group. The differences in secondary bile acid-producing bacteria were not statistically significant in this study. Mullish et al. [42] found that the BaiCD operon was not present in all bacteria with 7α-dehydroxylating ability, which has been considered an important process for secondary bile acid formation in faeces [13].

Metabolomics provides a qualitative and quantitative method of metabolite in analysis that can complete analysis along with microbiology. Metabolites (small molecules < 1500 Da) are cellular metabolism intermediates or end products, that can be produced directly by the host organism or can be derived from various other external sources, such as the diet, microbes, or xenobiotic sources [43]. Biological systems display complex and analytical limitations, and it is not possible to identify all the metabolites present in a specimen. Studies on metabolites and diseases indicate changes in diabetes [44], cardiovascular disease and heart failure [45, 46], autism [47] and anxiety [48]. As research progresses, metagenomic markers can be utilized for early disease diagnosis or cancer screening, and gut microbiota biology can indicate the effectiveness of cancer therapies and has predictive potential [49].

## Conclusion

In this study, gut microbiota analysis in the faeces showed that the abundances of *Weissella* and *Lactobacillus* were decreased, and those of *Bacteroides* and *Citrobacter* were increased in the CAP group. The increased abundances of *Bacteroides* and *Citrobacter* were positively correlated with CAPs, which indicated that changes in specific genera might be detrimental to intestinal health. In addition, metabolite detection showed changes in butyrate content, indicating that additional experiments are needed to investigate the function of butyrate in the intestinal environment. The increased concentration of *t10,c12-CLA* plays an important role in protecting the intestine. Analysis of the gut microbiota demonstrated carriage of operons producing metabolites, which indicated that additional functional operons might exist in the gut microbiota or intracellularly. Therefore, further studies focusing on lifestyle, diet and other factors in different populations are required to confirm the effect on intestinal health.

## Supplementary Information

**Additional file 1 Figure S1.** The alpha-diversity comparison of faeces between two groups, **A** Chao, **B** Ace, **C** Shannon and **D** Simpson index were present microbial community abundance and diversity. NS: None significantly differences. **Figure S2.** PCoA analysis: PC1 coordinates represent the main coordinate component that caused the largest difference in the sample, and PC2 represents the second coordinate component. **Figure S3.** Heatmap of Weighted UniFrac analysis. On top, the cluster tree presented sample phylogenetic relationships, the change of diversity ratio along with colour from blue to red.

## Data Availability

The datasets used and/or analysed during the current study are available from the corresponding author on reasonable request.
