# Supplementary material for: Bacteroides, butyric acid and t10,c12-CLA changes in colorectal adenomatous polyp patients
Source: Gut Pathog. 2021 Jan 12;13:1. doi: 10.1186/s13099-020-00395-0 (PMC7805033; doi:10.1186/s13099-020-00395-0)
Supplement: Supplementary file 1 — Additional file 1 Figure S1. The alpha-diversity comparison of faeces between two groups, A Chao, B Ace, C Shannon and D Simpson index were present microbial community abundance and diversity. NS: None significantly differences. Figure S2. PCoA analysis: PC1 coordinates represent the main coordinate component that caused the largest difference in the sample, and PC2 represents the second coordinate component. Figure S3. Heatmap of Weighted UniFrac analysis. On top, the cluster tree presented sample phylogenetic relationships, the change of diversity ratio along with colour from blue to red. [file 13099_2020_395_MOESM1_ESM.docx]

**Supplementary materials**


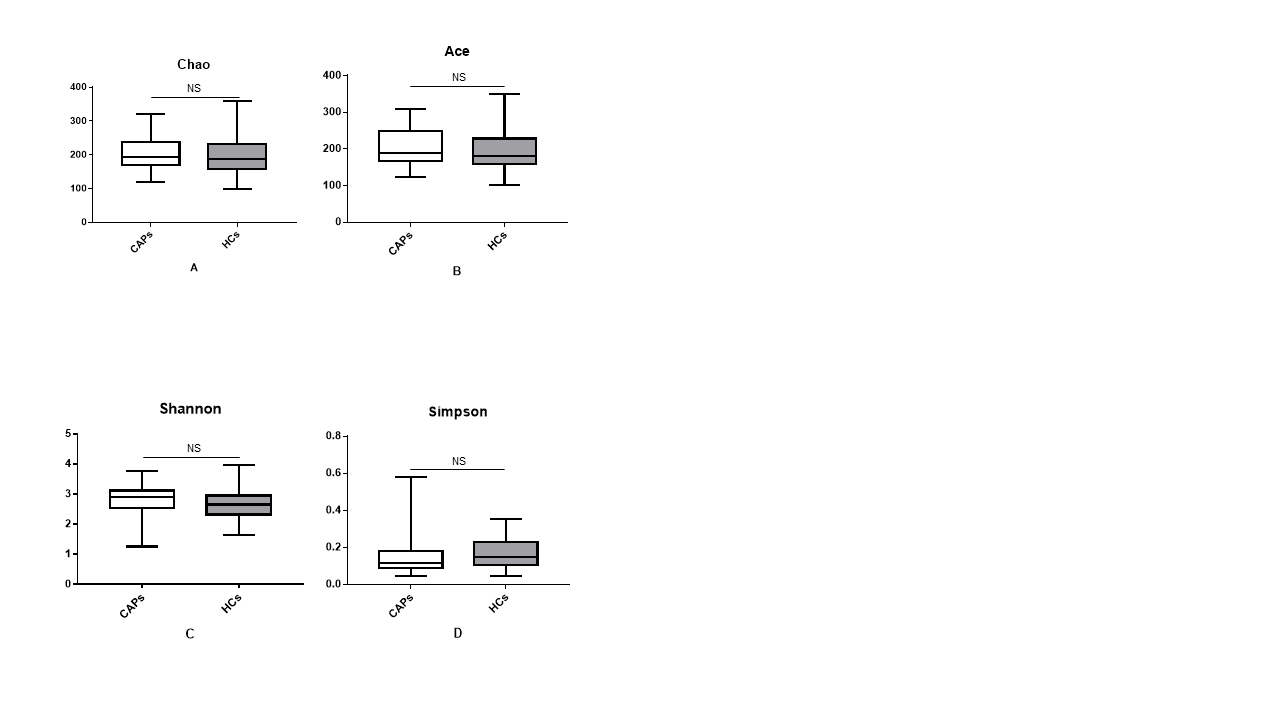


Figure S1. The alpha-diversity comparison of faeces between two groups, **A** Chao, **B** Ace, **C** Shannon and **D** Simpson index were present microbial community abundance and diversity. NS: None significantly differences.


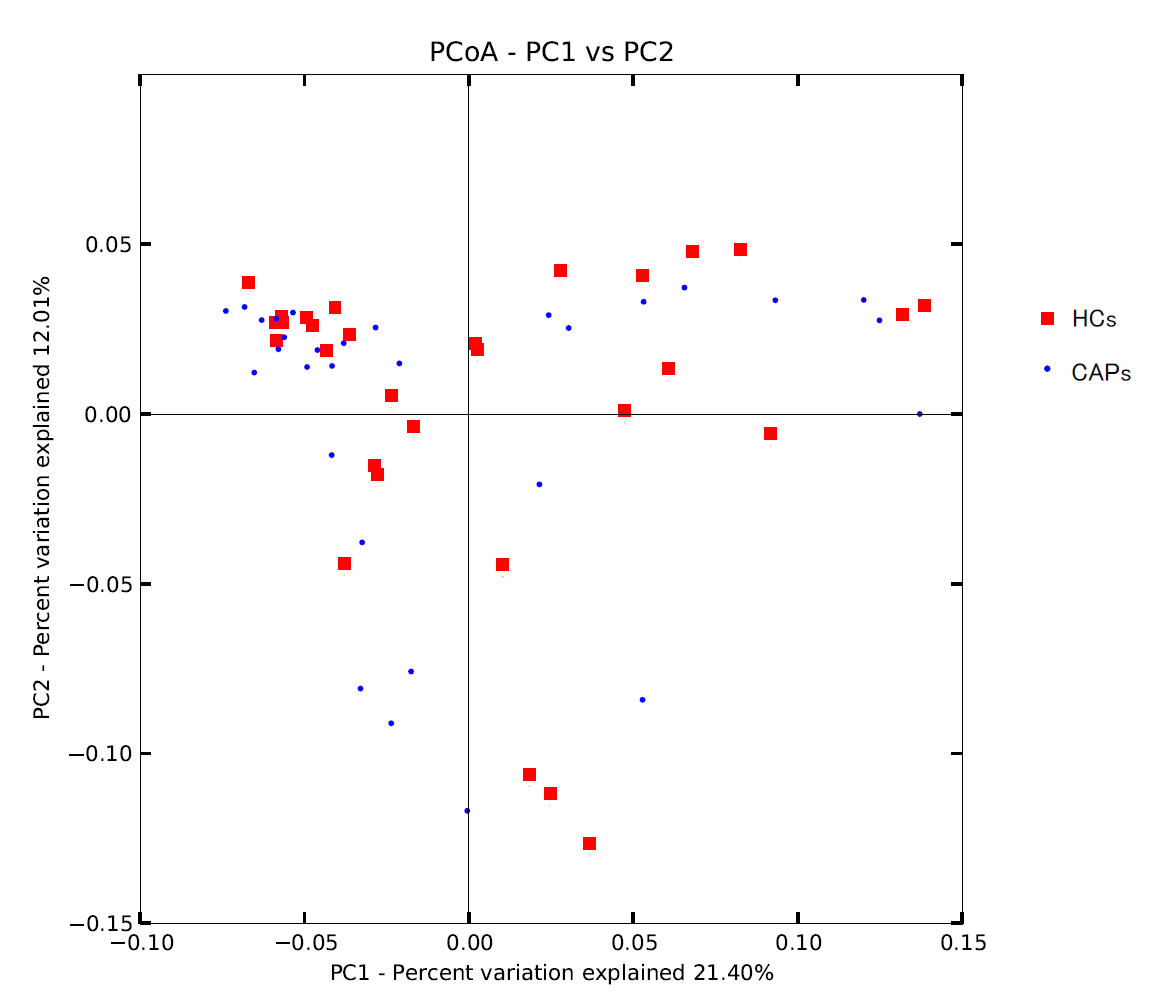


Figure S2. PCoA analysis: PC1 coordinates represent the main coordinate component that caused the largest difference in the sample, and PC2 represents the second coordinate component.


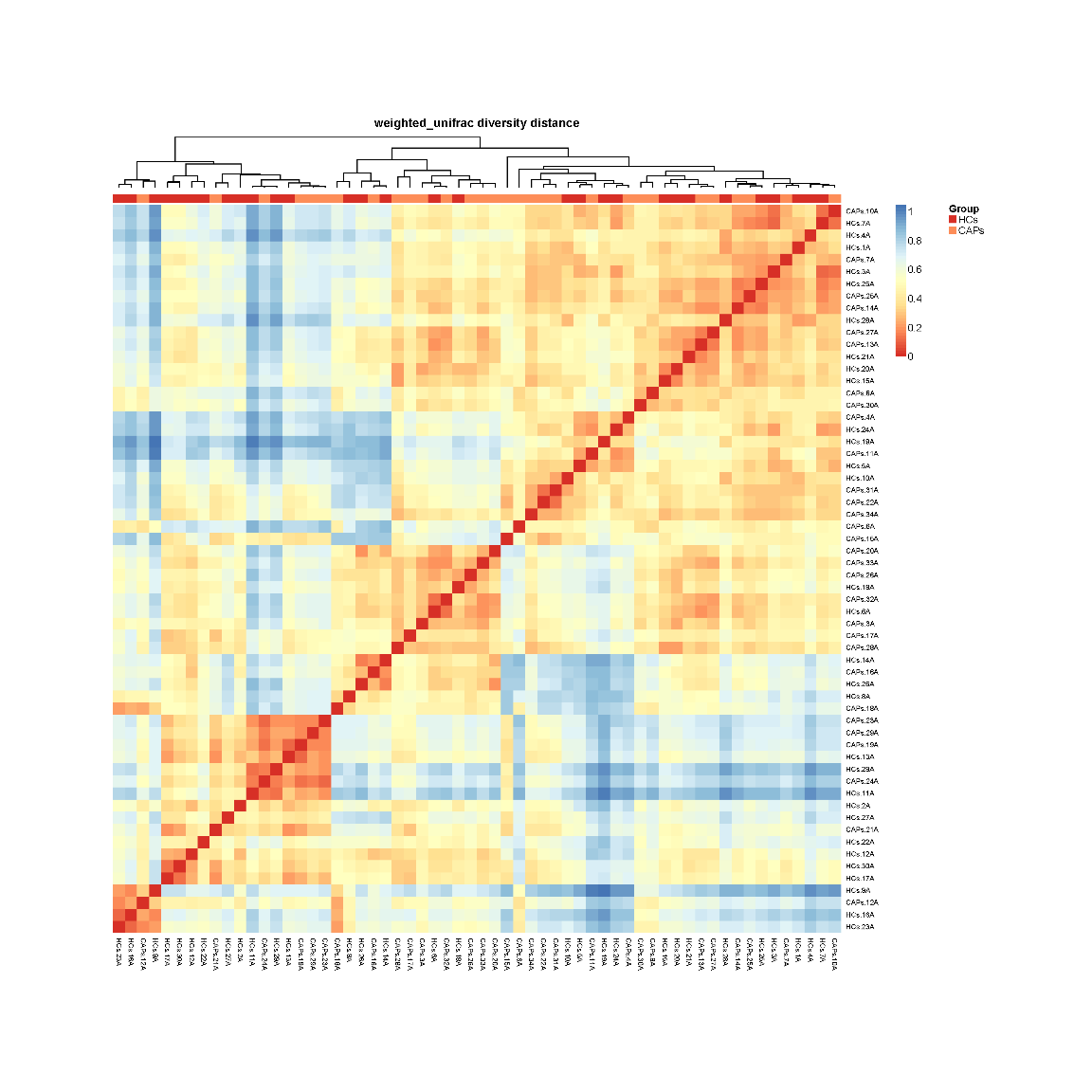


Figure S3. Heatmap of Weighted UniFrac analysis. On top, the cluster tree presented sample phylogenetic relationships, the change of diversity ratio along with colour from blue to red.
